# Flourishing at work: Psychometric properties of the Polish version of the Workplace PERMA-Profiler

**DOI:** 10.1371/journal.pone.0319088

**Published:** 2025-05-16

**Authors:** Paweł Fortuna, Agnieszka Czerw, Barbara Ostafińska-Molik, Agata Chudzicka-Czupała

**Affiliations:** 1 The John Paul II Catholic University of Lublin, Department of Experimental Psychology, Lublin, Poland; 2 University SWPS, Faculty of Psychology and Law, Interdisciplinary Center for Social Activity and Well-being Research FEEL & ACT WELL, Poznań, Poland; 3 Jagiellonian University, Institute of Education, Laboratory of Health Pedagogy, Laboratory of Pedagogical Diagnoses, Cracow, Poland; 4 University SWPS, Faculty of Psychology Interdisciplinary Center for Social Activity and Well-being Research FEEL & ACT WELL, Katowice, Poland; The John Paul II Catholic University of Lublin, POLAND

## Abstract

The main purpose of this study was to investigate the validity of the Polish version of the Workplace PERMA-Profiler (WPP). Our work was guided by Martin Seligman’s perspective on positive psychology, emphasizing well-being as its central theme, flourishing as the gold standard for measuring well-being, and the ultimate goal of increasing flourishing. According to his PERMA model, flourishing encompasses five key elements: positive emotions, engagement, relationships, meaning, and accomplishment. The WPP is a tool specifically designed to measure this construct in the workplace context. Polish workers completed online surveys at the initial measurement (N = 1070). In addition, a group of working adults (N = 66) took part in a survey with a repeated measure diagnosing measurement stability. Flourishing, perceived stress, and work satisfaction were measured for comparisons of convergent validity, while zero-sum belief was measured for divergent validity. The reliability indices of the Polish version of the WPP met the minimum reliability requirements, and the Confirmatory Factor Analysis indicated that the five-factor model (contrasted to the single-factor model) achieved the desired goodness-of-fit properties. The WPP also demonstrated convergent validity through strong interrelationships with related constructs and strong stability. Obtaining satisfactory psychometric properties of the Polish version of the WPP enabled the conduction of additional type of exploratory analyzes focused on the relationship between workplace flourishing and the efforts aimed at building employee well-being undertaken by the organization where the respondents work. The study revealed a positive, moderate relationship between employees’ flourishing and the evaluation of organizational practices for increasing employee well-being.

## Introduction

Measurement of well-being, happiness or flourishing [1] in the context of professional work is becoming a key element of human resources management in organizations today [2]. Employee well-being is often broadly understood as encompassing their overall physical, mental, and emotional health, and it serves as an overarching term that includes both happiness and flourishing. Happiness refers to the subjective, hedonic aspect of well-being, primarily associated with feelings of joy, satisfaction, and contentment. In contrast, flourishing can be viewed as a particularly high level of well-being, incorporating both the hedonic aspect (happiness) and the eudaimonic aspect (such as meaning and personal growth) [3]. The starting point for our text is Martin Seligman’s book [1] “Flourish: A visionary new understanding of happiness and well-being”, in which he presents the PERMA model based on five key elements. Admittedly, in the text we will often use the term well-being (because the sources we cite use this very term) but our key variable is treated as flourishing.

Many organizations are already aware that taking care of well-being in the workplace has benefits for the organization. Happy employees are effective, creative and committed to work [4,5]. Employees who feel appreciated and cared for are also less likely to change jobs [6]. Employee well-being also reduces absenteeism and presenteeism [7,8], which are often a serious organizational cost. It is also worth noting that organizations that care about the well-being of their employees build a positive image as an employer. This, in turn, attracts talent and increases the company’s competitiveness on the labor market.

Methods for measuring well-being, happiness or flourishing in the workplace can vary greatly. Organizations use their own short surveys measuring job satisfaction, use external companies implementing large multi-dimensional projects or use specific psychological tools based on one of many theories of well-being. One such theory is the PERMA model [1] and its application to the context of professional work [9].

The PERMA model, created by Seligman, one of the pioneers of positive psychology, is a framework for understanding flourishing, understood as a state in which individuals experience high levels of psychological well-being and realize their full potential [1]. The acronym PERMA refers to five key elements that Seligman believes are necessary to achieve optimal levels of flourishing:: positive emotions, engagement, relationships, meaning, accomplishment. The model is attractive and willingly applied in various contexts, among others because it combines two philosophical perspectives on defining well-being in psychology [10,11]. There are hedonistic (e.g., emotions) and eudaimonistic elements (e.g., meaning). Based on this model, a questionnaire was created to measure general well-being or actually flourishing – PERMA Profiler (PP) [12] and, slightly later, well-being in the context of professional work – Workplace PERMA Profiler (WPP) [9].

The original WPP is composed of 15 items referring to the key five dimensions (PERMA - 3 items are dedicated to each) and two additional scales: negative emotions (N), health (H) (3 items each), as well as single items regarding overall happiness at work (Hap) and sense of loneliness at work (L) [9]. The person responds to each item on a 10-point scale. The authors [9] of the questionnaire propose that in the individual diagnosis of a specific person, a flourishing profile consisting of five basic dimensions and two additional scales: PERMA + N + H, should be presented. Additionally, an overall well-being index can be calculated as the sum of five basic dimensions and a single item regarding overall happiness at work (16 items: PERMA + Hap). This strategy for calculating the result is often used in analyzes presented in numerous articles [e.g., 13, 14]. It is also possible to add information about the result for the item diagnosing the feeling of loneliness (L) which, however, is rarely reported in scientific articles. It is worth emphasizing that although the WPP is designed to measure flourishing, its overall indicator is based on the question *“Overall, how happy do you feel?”* and is referred to as “overall happiness” (Hap). These are the original terminological choices used by the authors of the WPP, which also highlights the broad approach this method takes in assessing the quality of functioning at work.

The primary aim of the research presented in this article was to investigate the validity of the Polish version of the WPP, which constitutes a natural step towards extending the analysis of employee flourishing within the PERMA model. The studies not only determined the structure of the PERMA model in a Polish sample of working individuals but also provided data offering a better insight into the nature of flourishing in the work context. This was achieved by capturing the relationships between well-being at work, as measured by this questionnaire, and other positive (job satisfaction) and negative (work stress) feelings at work. The recent experience of the coronavirus pandemic and the fact that Poland is one of the countries that received the largest number of refugees from Ukraine as a result of the Russian-Ukrainian war are important for the condition of Polish companies [13]. From December 2022 Eurostat publishes a new indicator on annual average salaries in the EU. According to these statistics, Poland ranks at the bottom, while the workforce in Poland is characterized by highly educated and skilled employees [14]. This can lead to considerable frustration among Polish workers. It is worth emphasizing that for the sake of the current and future prosperity of the organizations, it is important to adopt a human-centered approach to human resources and take care of the workers. So far, research on employee well-being has been conducted mainly from a hedonistic perspective, and the tools used have been limited to measuring the experience of emotions in professional situations and the determinants of job satisfaction, ignoring the eudaimonistic approach, related to the pursuit of goals, the value and meaning of work [15], especially in a broader flourish perspective, as enabled by the PERMA model. Adapting this tool can help to fill the existing gap in research, and it may also be useful in practice to systematically monitor employee flourishing.

Research confirms the existence of a cultural norm of negativity and complaining in Poland, which dictates the expression of dissatisfaction in a certain range of topics [16]. When the speaker complains, the most appropriate and adequate behaviour is to respond by complaining, which makes him feel understood by us, and we are perceived as wise and capable of forming close and deep relationships with others. This is one of the reasons why it is particularly important to conduct in Poland research on well-being in terms of positive psychology, an element of which is a sense of happiness resulting from and experienced at work. There is a need for a tool that promotes and perpetuates a different view of work and allows for cultural comparisons.

Additionally, the research explored the connection between employee well-being and the perception of one’s workplace as caring about the well-being of its employees. The presentation of the research process and results was preceded by a review of the national adaptations of the WPP, as well as modifications and an overview of research conducted using this questionnaire.

## Overview of the international adaptations of Workplace PERMA Profiler and modifications

Since the development of the original, American WPP questionnaire [9], adaptations in other languages of this tool have begun to appear in many countries. A review of websites and publications resulted in seven national versions of this questionnaire. Some of them are only translations, without a complete cultural adaptation procedure. Only tree are questionnaires published in scientific articles and meet the conditions for cultural adaptation. A summary of the national versions of the WPP is provided in Table 1. Listed here are only those versions of the questionnaire that have been directly published on https://www.peggykern.org/questionnaires.html and in articles presenting national adaptations. There might be more translations of this questionnaire that are used in individual studies.

**Table 1:** National versions of the Workplace PERMA-Profiler.

Country/language	Model tested	Results/structure	Participants	References
France/French	unknown	unknown	unknown	Translation available at https://www.peggykern.org/questionnaires.html
Germany/German	unknown	5 factors PERMA	mixed sample of 342 people: employees from the gastronomy sector (60%), employees from multiple sectors (40%); sample size unknown	Translation and basic information about sample available at https://www.peggykern.org/questionnaires.html
Japan/Japanese	Confirmatory Factor Analysis (CFA)	5 factors PERMA + N + H + L	310 workers registered as respondents of an Internet survey company, Macromill, Inc 21	[17]
South Korea/Korean	CFA	5 factors PERMA + N + H + L	316 workers, age: 20-over 50 years; gender: Men = 49%, Women = 51%	[18]
Portugal/Portuguese	unknown	unknown	unknown	Translation available at https://www.peggykern.org/questionnaires.html
Mexico/Spanish	unknown	5 factors PERMA + N + H + L	unknown	Translation available at https://www.peggykern.org/questionnaires.html
China/Chinese	CFA	5 factors PERMA + N + H + L	312 workers; age: M = 40.4; SD = 12.2; gender: Men = 56%, Women = 44%	[19]

It is also worth noting that some authors choose not to adapt a ready-made questionnaire, but rather create their own tools based on the theoretical PERMA model. An example is a Hungarian tool [20] consisting of six dimensions: (1) negative aspects of work; (2) meaning; (3) positive relationships; (4) engagement; (5) positive emotions – optimism; (6) accomplishment.

Still another approach is to extend the original model with additional components. Donaldson and Donaldson [21] propose the PERMA+4 framework. The proposed model traditionally includes five basic components but additionally includes: (1) physical health – a combination of high levels of biological, functional and mental health resources (this dimension is also present in the full version of the WPP) [9]; (2) mindset – an optimistic, future-oriented outlook on life, in which challenges and setbacks are viewed as opportunities for growth; (3) work environment – the quality of the physical work environment (such as temperature, lighting, etc.) but also a positive psychological climate; (4) economic security – perceived security and financial stability necessary to meet individual needs.

## A brief review of research conducted using the Workplace PERMA-Profiler

It is quite surprising how little research has been conducted using the WPP. Since the inception of this questionnaire, there have been three articles presenting national adaptations that examined not only the structure, but also the convergent validity of new versions of the tool.

The Japanese adaptation process of the WPP [17] showed moderate and strong correlations with work satisfaction (0.60 ≤ r ≤ 0.76) and weaker with life satisfaction (0.19 ≤ r ≤ 0.32). Other work context variables studied were also positively related to the WPP: work engagement (0.69 ≤ r ≤ 0.82), support from supervisors (0.32 ≤ r ≤ 0.53), support from colleagues (0.32 ≤ r ≤ 0.60), work performance (0.48 ≤ r ≤ 0.73). Choi and colleagues [18] in preparing the Korean version of the WPP examined how workplace flourishing is related to several constructs of functioning at work. They showed moderate to strong positive correlation with work engagement (0.52 ≤ r ≤ 0.81) and professional efficacy (0.47 ≤ r ≤ 0.64). On the other hand, the WPP showed a moderate negative correlation with the exhaustion (-0.50 ≤ r ≤ -0.19) and cynicism (-0.51 ≤ r ≤ -0.37), occupational stressors (-0.59 ≤ r ≤ -0.30), and stress responses (-0.62 ≤ r ≤ -0.30). A Chinese study [19] found significant associations with the Checklist Individual Strength (CIS) diagnostic on several dimensions of fatigue: fatigue severity, concentration problems, reduced motivation, reduced physical activity level. All the relationships of the overall CIS index with individual PERMA components are negative, at the average level (0.31 ≤ r ≤ 0.56). More varied relationships are found at the level of individual CIS components. The weakest relationship with well-being is shown by physical activity, which does not correlate at all with engagement and meaning, and the correlation with the others is weak (0.18 ≤ r ≤ 0.29).

Other studies [21] have confirmed some of these relationships. Among other things, they showed positive relationships with organizational citizenship behavior (0.23 ≤ r ≤ 0.40), positive work role performance understood as: individual proficiency, team proficiency, organizational proficiency, individual adaptivity, team adaptivity, organizational adaptivity, individual proactivity, team proactivity, organizational proactivity (0.24 ≤ r ≤ 0.71), and negative relationships with turnover intentions (-0.34 ≤ r ≤ -0.61). Another study [22] aimed to examine the relationship between teachers’ psychological resources, especially taking into account the concept of psychological capital, and perceived well-being in the workplace. The results indicated a significant relationship (0.34 ≤ r ≤ 0.61) between workplace well-being (workplace PERMA Profiler) and personal psychological resources, particularly hope (r = 0.30) and optimism (r = 0.49).

Another aspect was pointed out in a study of public relations employees [23]. Authors pointed to interesting differences in well-being at work between employees of different genders. They found that women experience greater well-being than men in the areas of engagement and positive relationships, as well as an assessment of their health (0.001 ≤ *p* ≤ 0.04). However, this is a significant difference in only two of the five core dimensions of well-being and one of three additional ones. In addition, this study found no association of well-being with age or seniority. It is intriguing to see whether this pattern of association of well-being at work with employees’ gender and age has a more persistent pattern.

The PERMA model in the context of work also provides inspiration for designing interventions that build employee well-being. Beacham and colleagues [24] conducted a pilot study of such an intervention in a group of health care workers. The intervention was based on exercises derived from positive psychology and, in particular, tasks based on mindfulness, acceptance and commitment therapy. The results obtained are promising. The intervention raised the overall index of well-being at work, and this was especially true on the scale of positive emotions and engagement. A similar problem was taken up by Wingert and colleagues [25] on a group of working students. This time the intervention involved an 8-week mindfulness-based strengths practice. In this study it was found that the students involved in the intervention also had significantly higher overall well-being scores at work. In addition, they had statistically significantly higher scores on the scales of engagement, meaningfulness and health both compared to the pre-intervention measure and compared to the control group.

Measurement using the WPP questionnaire is not without some controversy. Research conducted in different countries and on different samples sometimes leads to the conclusion that the structure of the questionnaire is not fully confirmed and perhaps some modifications to this tool should be made [26,27]. This makes it even more important to confirm the structure of this questionnaire again, this time on a Polish sample of working people.

While designing this study, based on a review of the literature on the subject, the following hypotheses were formulated:

H1: According to the original PERMA theory, the five-factor model is expected to be the most suitable for the Polish version of the WPP.
H2: The Polish version of the WPP is expected to demonstrate convergent validity through strong interrelationships with related constructs such as flourishing in the perspective of personal life, job satisfaction and work stress.
H3: The Polish version of the WPP will exhibit high test-retest reliability, indicating stable measurement of well-being over time among Polish employees.

Considering the primary aim of measuring well-being with the Polish version of the WPP, the research was extended to include one additional aspect: the relationship between employee well-being and the perception of the workplace as caring about the well-being of its employees. This analysis was exploratory in nature; therefore, only research question was formulated:

Q1. Is employee well-being related to the perception of one’s workplace as caring about the well-being of its employees?

## Method

### Procedure

The validation study was conducted from May 2023 to March 2024 and comprised three phases: the translation of the questionnaire (May 27, 2023), a study verifying the structure and validity (July 5–25, 2023), and an assessment of measurement stability (March 1–10, 2024). Individuals involved in the main part of study were enlisted through the professional research firm and university research system one of the authors of this article. Survey participants were tasked with fulfilling online surveys accompanied by a concise explanation regarding the scholarly nature of the research. Respondents who opened the link to the survey first read the information about the study and agreed to voluntary and anonymous participation in the study. Continuation of the study was possible only after accepting the required consent. Test–retest reliability was investigated using the longitudinal data two weeks after follow-up. The studies were approved by the Research Ethics Committee of University SWPS (approval no. 2023–168).

## Sample

The study comprised 1070 participants (M_age_ = 37.47, SD_age_ = 11.04); 64.5% of whom were female (M_age_ = 36.05, SD_age_ = 10.75), 35.2% were male (M_age_ = 40.16, SD_age_ = 11.04), 0.1% identified their gender as other than the aforementioned categories (N = 1; age = 23), and 0.2% opted not to respond to this inquiry (N = 2; M_age_ = 23.50; SD_age_ = 3.54). In Poland there has been an overrepresentation of women relative to men for many years, and this applies to the age group over 40 [28]. Because the average age of men in our sample is just about 40 years old, this fact, according to the authors, can largely explain such a disproportion. The overall work experience averaged M = 15.16 years (SD = 10.06), while the average tenure within the current organization was M = 6.85 years (SD = 7.73). A significant proportion of individuals were employed in corporations with a workforce exceeding 250 individuals (39.4%), followed by companies with 51–250 employees (22.6%), 10–50 employees (24%), and 1–9 employees (13.9%). The majority of respondents worked in private sector organizations (70.9%), with a notable representation in state-owned enterprises as well (23.7%), while other organizational affiliations constituted only 5.3%. The predominant contractual arrangement was full-time or part-time employment contracts (79.1%), with alternative forms constituting the remaining 20.9%. In terms of educational qualifications, the majority held higher education degrees (Bachelor’s degree: 13.8%, Master’s degree: 47.5%), while the sample included 0.6% with primary education, 2.1% with vocational training, 10% with vocational education and training, 15.4% with secondary education (high school), 8.7% with post-secondary education, and 2% with a third-degree higher education (Doctorate).

### Measures

#### Workplace PERMA-Profiler (WPP).

The Polish version of the WPP was used to measure flourishing at work. The scale consists of 23 items that refer to the five-factor the five-factor PERMA model [1]: positive emotions, engagement, relationships, meaning and achievements (3 items each), as well as the level of general happiness at work (1 item) and negative emotions (3 items), health (3 items) and loneliness (1 item). Item examples: How often do you feel you are making progress towards accomplishing your work-related goals?; At work, how often do you feel joyful? How often do you achieve the important work goals you have set for yourself? Each WPP factor score was calculated as the average of the item ratings. The overall well-being score at work was calculated as the average of the 15 items and happiness (1 item). Respondents rated all items on an 11-point Likert-type scale (from 0 to 10). The response scales for individual items varied depending on the way the items were worded in the questionnaire, but always on a 11-point scale. Examples of extreme answers are given here: 0 – *terrible*…. 10 – *excellent*; 0 – *never*…. 10 – *always*; 0 – *not at all*…. 10 – *completely*. The answers are the same as in the original WPP scale.

Following approval from the scale’s author, two skilled English speakers, operating independently, undertook the translation of the instrument into Polish. Then, the WPP items were compared with the Polish translation of the PERMA-Profiler scale already used in Polish studies [29]. Due to the high similarity of items between the WPP and the original PP, we determined that the back-translation procedure was unnecessary. The content of the items in the WPP remains identical to the original, apart from minor modifications such as adding phrases like “in the workplace” or “at work” to reflect the specific context. The original PP had already undergone a rigorous back-translation process under the supervision of Butler, Kern and Kossakowska as part of its cultural adaptation into Polish. This validated translation is publicly available and widely recognized as reliable [30]. We therefore decided to use this particular earlier adaptation of the tool. Our decision aligns with practices in cross-cultural research, where validated translations of closely related instruments are adopted without a repeated back-translation, particularly when only minor contextual adjustments are introduced [31–33].

The Polish version of the WPP prepared in this way and the original English version of the WPP were tested by 30 teachers (22 women) of English, aged 28–53, who completed the scale during a conference at a time interval of five hours. The teachers were participants at an education conference and voluntarily completed the questionnaires after giving verbal consent to participate in the study. The t-Student test results for individual items showed that there were no significant differences between responses to individual the individual WPP items (p > .05).

The subsequent questionnaires were used to check the convergent and discriminant validity of the Polish version of the WPP. The PP, Perceived Stress Scale and Work Satisfaction Scale were considered indicators of convergent validity. The Zero-Sum Game Belief Questionnaire is an indicator of discriminant validity.

#### The PERMA-profiler.

(PP) designed by Butler and Kern [12] in the Polish translation by Kossakowska [30] was used to assess flourishing based on the five-factor PERMA model [1]. Item examples were: How often do you become absorbed in what you are doing?; To what extent do you receive help and support from others when you need it?; How often are you able to handle your responsibilities? As in the WPP participants responded to 23 items, providing answers on similar scales. The reliability coefficient for subscales ranged from *α* = .76 to *α* = .92.

#### Perceived Stress Scale.

(PSS-10) designed by Cohen, Kamarck and Mermelstein [34], in the Polish adaptation by Chirkowska-Smolak and Grobelny [35], contains 10 statements. Item examples: In the past month, how often have you been upset because something unexpected happened at work?; During the past month, how often have you felt that difficulties at work have multiplied to the point that you cannot overcome them? The respondents’ task was to respond to them on a Likert scale, where 1 meant “never” and 5 – “very often”. The reliability coefficient in original research was high *α* = 0.82.

#### Work Satisfaction Scale.

(WSS) designed by Zalewska [36], inspired by the very popular questionnaire *The Satisfaction With Life Scale* by Diener and colleagues [37]. It is a five-item questionnaire treating job satisfaction as a comprehensive, complex phenomenon. Item examples: In many ways, my work is close to perfect; So far, I’ve managed to achieve what I wanted at work. The examined person evaluates his/her work on a 7-point Likert scale of agreement (1 – *strongly disagree*,..., 7 – *strongly agree*) based on personal criteria, not imposed by the measurement method. The reliability coefficient in original research was high (*α* = .92).

#### The zero-sum game belief questionnaire.

Designed by Różycka and Wojciszke [38] measures the social belief in the hidden assumption that one person’s gain or success is only possible at the expense of another person’s loss or failure. This is a general variable, independent of the domain of professional work. This belief is typical of people who would like to gain something or are afraid of losing something, as well as for people dealing with economics where the basic law of the market is a limited amount of resources that require competition. The questionnaire consists of 12 items constituting one dimension. Item examples: Successes for some are almost always failures for others; In most situations, the interests of different people are in conflict Each question is answered on a 7-point Likert scale of agreement (1 – *strongly disagree*,..., 7 – *strongly agree*). The authors of the questionnaire indicate satisfactory internal consistency (alpha’s from 0.67 to 0.84) obtained in various samples.

#### Perceived care for employee well-being in the organization (firm well-being).

The subjects were asked two questions about the employee’s perceived organizational measures taken to promote well-being at work. The first was: rate how much your company ensures that employees experience happiness (well-being, health) while doing their jobs (1 – *not at all*,..., 5 – *definitely yes*). The second was an open-ended question: indicate the specific actions your company takes to take care of the well-being and health of employees (leave blank if you don’t see such actions).

### Statistical analyses

A sequence of psychometric examinations was executed to confirm structural validity of the WPP, encompassing inter-item correlation analysis (utilizing Pearson’s r), reliability analysis (involving Cronbach’s alpha, and McDonald’s omega), Confirmatory Factor Analysis (CFA), and ultimately, analyses pertaining to validity. The analyzes included the single-factor model (the 15 PERMA WPP items will load on a single workplace well-being factor) and the five-factor model (the 15 PERMA WPP items will load on five separate PERMA factors, where the factors are inter-correlated but treated as separate constructs).

All statistical analyses were carried out utilizing the R program [39] and RStudio [40], employing predominantly (in alphabetical order): corrplot [41], dplyr [42], haven [43], Hmisc [44], lavaan package [45], mvnTest [46], PerformanceAnalytics [47], psych [48], RColorBrewer [49], and semTools [50].

## Results

### Reliability assessment

In the first step, we computed the correlation coefficients between the 15 PERMA WPP items (Table 2). Most correlation coefficients were maintained at moderate and high positive levels.

**Table 2:** Correlation matrix for the 15 PERMA WPP items.

	P1	P2	P3	E1	E2	E3	R1	R2	R3	M1	M2	M3	A1	A2	A3
**P1**	1														
**P2**	.82	1													
**P3**	.81	.82	1												
**E1**	.51	.51	.51	1											
**E2**	.74	.74	.74	.58	1										
**E3**	.44	.44	.44	.35	.50	1									
**R1**	.59	.60	.59	.34	.49	.29	1								
**R2**	.64	.65	.64	.37	.53	.32	.67	1							
**R3**	.64	.64	.64	.36	.53	.32	.66	.71	1						
**M1**	.64	.64	.64	.49	.71	.42	.45	.49	.49	1					
**M2**	.71	.71	.71	.54	.78	.47	.50	.54	.54	.73	1				
**M3**	.69	.70	.69	.53	.76	.46	.49	.53	.52	.71	.79	1			
**A1**	.64	.64	.64	.47	.69	.41	.46	.50	.50	.63	.69	.68	1		
**A2**	.58	.59	.58	.43	.63	.38	.42	.46	.45	.57	.63	.62	.65	1	
**A3**	.34	.35	.34	.26	.37	.22	.25	.27	.27	.34	.38	.37	.39	.35	1

Note: P = Positive emotion, E = Engagement, R = Relationships, M = Meaning, A = Accomplishment

In the second step, we tested the baseline means, standard deviations and min-max of the PERMA WPP items. The indices used for relability of PERMA subscales were Cronbach’s alpha [51] and Omega total coefficient [52] (see Table 3).

**Table 3:** Mean scores and Min-Max of the 15 PERMA WPP items and reliability of PERMA subscales (N = 1080).

Factors	Baseline Mean (SD)	Min-Max	Cronbach’s alpha (α)	McDonald’s omega (ω)	Mean within-scale correlation	Mean inter-item correlations
Positive emotion			.93	.93	.82	
P1	6.03 (2.61)	0-10				.65
P2	6.41 (2.93)	0-10				.65
P3	6.25 (2.59)	0-10				.66
Engagement			.74	.75	.49	
E1	6.92 (3.11)	0-10				.48
E2	5.95 (2.29)	0-10				.65
E3	5.83 (2.19)	0-10				.43
Relationships			.86	.86	.68	
R1	6.92 (2.80)	0-10				.50
R2	6.16 (2.52)	0-10				.55
R3	6.61 (2.77)	0-10				.58
Meaning			.90	.90	.75	
M1	7.33 (3.31)	0-10				.58
M2	6.73 (2.68)	0-10				.64
M3	6.29 (2.45)	0-10				.65
Accomplishment			.73	.77	.46	
A1	6.51 (2.83)	0-10				.60
A2	6.59 (2.85)	0-10				.55
A3	7.97 (4.40)	0-10				.39

The goodness-of-fit criteria assumed in the reliability analysis were Cronbach’s alpha > .80 [53] and McDonald’s Omega > .70 [54]. The reliability indices for positive emotions, relationships, and meaning met minimum reliability requirement. Although for the engagement and accomplishment, a satisfactory level of Cronbach’s alpha was not achieved, it was attained for McDonald’s omega, which appears to be a more robust measure for multidimensional variables.

### Structural validity

In the subsequent phase of our investigation, we assessed the conformity of the model with the data, exploring both the single-factor model and the five-factor model. Each model was evaluated based on various criteria gauging goodness-of-fit, including the significance of the chi-square test along with the chi-square/df ratio, the Root Mean Square Error of Approximation (RMSEA), the Comparative Fit Index (CFI), the Tucker-Lewis Index (TLI), and the Standardized Root Mean Square Residual (SRMR). Our benchmarks for an acceptable fit encompassed a chi-square/df ratio < 3, as per [55], CFI and TLI values equal to or surpassing.90 [56,57], and RMSEA and SRMR values below.08 [55,57]. Alternatively, indicators pointing to an exemplary fit included a chi-square/df ratio below 2 [55], a CFI value equal to or exceeding.95 [57,58], and RMSEA and SRMR values lower than.05.

In light of the outcomes derived from tests evaluating normality, such as Mardia’s multivariate skewness and kurtosis tests [59], the Henze-Zirkler Test [60], and the Doornik-Hansen Test [61], parameter estimation was carried out using the Maximum Likelihood with Robust Standard Errors (MLR) method. This selection was prompted by the absence of multivariate normality, as recommended by Muthen and Muthen [62]. MLR, a recalibration-centric estimation technique tailored for non-normally distributed data, yields standard errors and a chi-square test, distinguishing it from comparable methodologies [63].

To compare the models in the 5-factor solution with the model in the 1-factor solution, we followed two steps. First, we conducted a unidimensionality analysis on the 1-factor solution to verify whether introducing a multi-factor solution was necessary. The unidimensionality of the scale was confirmed (u = 0.92, tau = 0.93, alpha = 0.95, ECV = 0.88). However, secondly, we compared the Bayesian Information Criterion (BIC) for both models, which turned out to favor the 5-factor solution (BIC = 61127.44) compared to the 1-factor model (BIC = 62258.95). We consider this sufficient motivation to adopt the 5-factor model (not to mention the fit indices, as well as the content validity of the factors and their theoretical significance). The analysis of goodness-of-fit indices showed that in the case of the single-factor model satisfactory parameters (CFI, TLI, RMSEA, SRMR; see Table 4) were not obtained to accept this solution. However, the analysis of the five-factor model allowed us to achieve the desired goodness-of-fit properties (see Table 5 for factor loadings and standard errors).

**Table 4:** Structural validity: comparison of model fit indices for 1-factor and 5-factor models.

Model fit	1-factor	5-factor
scaled χ2 (df)	1238.61(90)	440.70(80)
robust CFI	.867	.960
robust TLI	.844	.947
robust RMSEA (95% CI)	.133 (.126,.139)	.077 (.070,.085)
SRMR	.056	.032

1-factor model vs. 5-factor model: Δχ2(df) = 797.91(10)***

Estimator: MLR. Note. RMSEA = root mean square error of approximation; CFI = comparative fit index; TLI = Tucker-Lewis index; SRMR = standardized root mean square residual.

**Table 5:** Factor loadings of the 15 PERMA WPP items.

Items	Factor loadings	
	1-factor model (std. err.)	5-factor model (std. err.)
P1	.86 (.02)	.90 (.02)
P2	.86 (.02)	.91 (.02)
P3	.89 (.02)	.90 (.03)
E1	.59 (.04)	.63 (.03)
E2	.87 (.03)	.92 (.09)
E3	.52 (.04)	.55 (.08)
R1	.61 (.04)	.78 (.03)
R2	.69 (.03)	.85 (.03)
R3	.73 (.03)	.84 (.04)
M1	.76 (.03)	.81 (.02)
M2	.85 (.03)	.90 (.04)
M3	.86 (.03)	.88 (.05)
A1	.77 (.04)	.85 (.04)
A2	.70 (.04)	.77 (.04)
A3	.45 (.03)	.46 (.04)

In the 1-factor model, most items exhibited strong factor loadings (>.60), with the exception of a few items, such as E3 (λ = .52, SE = .04) and A3 (λ = .45, SE = .03), which had relatively low loadings. Notably, items within the “P” factor demonstrated the strongest loadings (λ range:.86–.89). Items in the “E” and “A” factors showed more variability in their loadings, with E3 and A3 falling below the commonly recommended threshold of.50, indicating weaker representation of the factor. The 5-factor model demonstrated generally improved factor loadings across all items, highlighting a better alignment with their respective latent factors. Items within the “P” factor remained strong (λ range:.90–.91), and loadings for the “E” factor showed a slight increase, particularly for E2 (λ = .92, SE = .09). Similarly, items within the “R,” “M,” and “A” factors also exhibited higher loadings compared to the 1-factor model, with notable improvements for items like R1 (λ = .78, SE = .03) and R2 (λ = .85, SE = .03). However, A3 continued to display a low loading (λ = .46, SE = .04), suggesting it may not adequately represent its latent factor.

**Fig 1:**
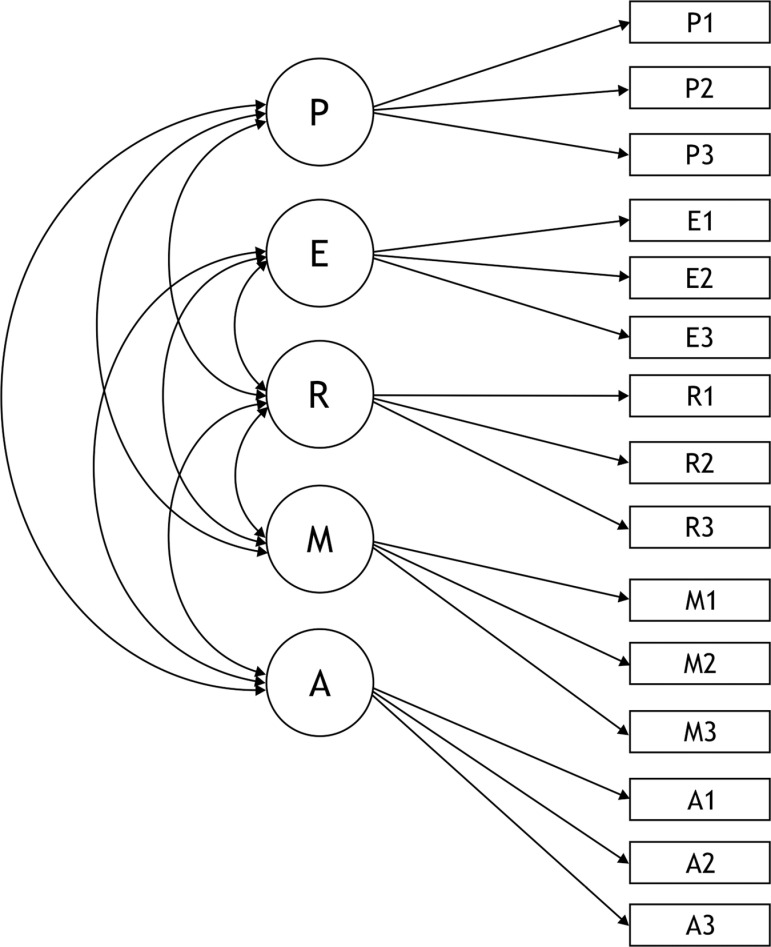
Five-factor PERMA model. PP = Positive Emotion; EE = Engagement; RR = Relationships; MM = Meaning; AA = Accomplishment. P1–P3, E1–E3, R1–R3, M1–M3, and A1–A3 represent the individual items corresponding to each scale.

The 5-factor model (Fig 1) was then used to verify sex and cultural measurement invariance.

We assessed the fit of each step in the measurement invariance analysis (configural, metric, scalar, and strict) using the chi-squared test alongside various fit indices such as CFI, TLI, RMSEA, and SRMR. It is worth noting that the chi-squared statistic is highly sensitive to minor model deviations, which may lack practical significance [58,64–66]. Consequently, fit indices are often considered more reliable for evaluating model fit in the context of measurement invariance [58]. In our analysis, acceptable model fit was determined using established thresholds: CFI and TLI ≥ .90 [56,57] and RMSEA and SRMR < .08 [55,57,67].

Our approach required meeting at least two out of three criteria (ΔCFI, ΔRMSEA, ΔSRMR) to confirm measurement invariance at each stage of the analysis. To examine sex measurement invariance, we first conducted a Confirmatory Factor Analysis (CFA) for the 5-factor model separately for men and women, ensuring adequate model fit within each subgroup of the Polish sample. Subsequently, we performed a standard measurement invariance analysis across the entire Polish sample, beginning with the configural invariance stage.

The 5-factor model exhibited acceptable fit for both men and women, meeting the established criteria of CFI and TLI ≥ .90, and RMSEA and SRMR ≤ .08 (see Table 6). Following the analysis of sex measurement invariance, the configural, metric, scalar, and strict invariance models all demonstrated acceptable fit according to the same thresholds (CFI and TLI ≥ .90, RMSEA and SRMR ≤ .08). During the metric invariance assessment, all cutoff criteria were satisfied: ΔCFI ≤ .005, ΔRMSEA ≤ .01, and ΔSRMR ≤ .025. Similarly, for the scalar and strict invariance stages, the criteria were successfully met: ΔCFI ≤ .005, ΔRMSEA ≤ .01, and ΔSRMR ≤ .005.

**Table 6:** Psychometric indicators for sex measurement invariance analysis.

Model	χ^2^	*df*	χ^2^/*df*	*p*	RMSEA	CFI	TLI	SRMR	Model comparison	Δχ^2^	Δ *df*	*Pr (>*χ^2^)	ΔRMSEA	ΔCFI	ΔSRMR	Decision
Male	211.09	80	2.64	<.001	.077	.961	.949	.035								–
Female	329.71	80	4.12	<.001	.079	.958	.945	.034								–
(1) Config.	541.58	160	3.38	<.001	.078	.959	.946	.034								–
(2) Metric	550.34	170	3.24	<.001	.076	.959	.949	.039	(1) - (2)	8.76	10	.550	-.002	.000	.005	Accept
(3) Scalar	572.62	180	3.18	<.001	.074	.958	.951	.040	(2) - (3)	22.28	10	.044	-.002	-.001	.001	Accept
(4) Strict	584.56	195	3.00	<.001	.073	.957	.954	.041	(3) - (4)	11.94	15	.091	-.002	-.001	.001	Accept

Estimator: MLR. Note. Config. = configural; Δχ2, Δ*df*, Pr (>χ2), ΔRMSEA, ΔCFI, and ΔSRMR denote the change in the chi-square value, degrees of freedom, the significance of these changes, changes in RMSEA, CFI, and SRMR respectively.

In the next step, a correlation analysis of latent variables was conducted in the five-factor model to ascertain the degree of interdependence among the variables constructing the model (see Table 7).

**Table 7:** Correlation coefficients in the five-factor model.

	F1 (P)	F2 (E)	F3 (R)	F4 (M)	F5 (A)
F1 (P)	1.00				
F2 (E)	.89	1.00			
F3 (R)	.84	.68	1.00		
F4 (M)	.87	.95	.71	1.00	
F5 (A)	.84	.89	.70	.91	1.00

*Note.* P = Positive emotion, E = Engagement, R = Relationships, M = Meaning, A = Accomplishment

The analysis provides information that the variables are highly or very highly correlated with each other. However, the matrix achieved convergence during the analysis, indicating that we do not face an issue of collinearity or Heywood-Case problem. The presented analyzes positively verify H1.

### Construct validity

The final phase of the analysis focused on examining construct validity, encompassing both convergent and discriminant aspects. PP, PSS-10 and WSS were employed to assess convergent validity, while The Zero-Sum Game Belief Questionnaire were utilized to evaluate discriminant validity (see Table 8).

**Table 8:** Correlation matrix for the convergent (PERMA Profiler, Perceived Stress Scale, Work Satisfaction Scale) and discriminant (The Zero-Sum Game Belief Questionnaire) validity of the Workplace PERMA-Profiler.

Variables	Mean (SD)	(WPP-P)	(WPP-E)	(WPP-R)	(WPP-M)	WPP-(A)	Overall
PP							
Positive emotion (P)	6.80 (1.77)	.61**	.45**	.49**	.51**	.53**	.60**
Engagement (E)	6.86 (1.64)	.58**	.64**	.44**	.58**	.58**	.65**
Relationships (R)	7.03 (1.80)	.51**	.37**	.54**	.43**	.45**	.53**
Meaning (M)	6.88 (1.92)	.59**	.50**	.47**	.59**	.56**	.62**
Accomplishment (A)	6.97 (1.65)	.57**	.51**	.48**	.57**	.66**	.64**
Overall happiness	6.90 (1.55)	.65**	.56**	.55**	.60**	.63**	.69**
Negative emotion	13.24 (6.60)	-.53**	-.23**	-.47**	-.40**	-.33**	-.45**
Health	19.46 (6.25)	.48**	.30**	.42**	.37**	.41**	.46**
Loneliness	3.81 (2.79)	-.37**	-.16**	-.44**	-.27**	-.23**	-.34**
PSS-10	31.38 (4.12)	-.30**	-.06	-.28**	-.19**	-.18**	-.24**
WSS	22.46 (6.70)	.80**	.65**	.65**	.74**	.67**	.81**
Zero-Sum Game Belief	3.44 (0.96)	-.13**	-.13**	-.12**	-.16**	-.15**	-.16**

*Note.* Overall = the average of the 15 PERMA items and the overall happiness item; PP – PERMA Profiler; PSS-10 – Perceived Stress Scale; WSS – Work Satisfaction Scale; WPP – Workplace PERMA-Profiler; **p < .001

The results indicate that all variables potentially serving the role of convergent validity – all PP subscales (PERMA, health, overall well-being: positively and negative emotions, loneliness: negatively) as well as work satisfaction (positively) and perceived stress (negatively) confirm this type of validity for the WPP method. The indicators for the zero-sum game as a discriminant disadvantage variable are statistically significant, but all of them are very low. In the light of the obtained results, H2 was confirmed.

### Test–retest reliability

The last step of the construction process was to check the stability of the measurement made by the Polish version of the WPP. One hundred working individuals were recruited for the study; and it’s worth noting that this was a separate sample from the one described earlier in the study that checked the structure of the questionnaire. The criterion for entry into the sample was seniority of at least 1 year. The subjects completed the WPP two weeks apart. Before the second measurement, brief interviews were conducted to control for possible changes (organization, position, responsibilities, in private life) that could significantly affect the measurement. Finally, data obtained from a group of 66 subjects with no lesions were analyzed. The characteristics of the group were as follows: gender (F = 36; M = 30); age (M = 36.56; SD = 12.95); 21 people in managerial and 45 non-managerial positions; 14 people with secondary education and less, and 47 with higher education. Correlations between the two measurements were found to be strong (P1-P2 = .75, E1-E2 = .77, R1-R2 = .66, M1-M2 = .85, A1-A2 = .74, Overall1-Overall2 = .85, Negative emotions = .77, Health = .81) and significant (p < .001), which positively verifies H3.

### Additional analysis: Workplace flourishing and perceived care for employee well-being in the organization

When checking the structure of the Polish version of the questionnaire, respondents were asked about practices supporting employee well-being in their organizations. This question was asked to check whether the introduction of such activities into organizational practices is actually related to the level of employee flourishing. The correlations between the perceived care for employee well-being in the organization (PC) and the WPP PERMA subscales were moderate (PC-P = .58, PC-E = .44, PC-R = .54, PC-M = .50, PC-A = .46) and statistically significant (p < .001). Thus, it can be said that research Q1 was answered confirming the relationship between employees’ flourishing and the organization’s actions.

In addition, it is worthwhile to provide a qualitative analysis of the statements of employees who indicated what initiatives their organizations are undertaking to enhance employee flourishing. We used a strategy modeled on grounded theory (one of the key ones in qualitative data analysis) to analyze the data: data review, open coding (as no categories were assumed beforehand) and identification of main themes [68]. Employee statements can be divided into several categories.

- Flexible time and form of work performance: e.g., 7-hour workday, additional fully paid days off, freedom to choose the days for work provided online, no restrictive control of working hours during the day, etc.
- Above-standard access to somatic and mental health support services: e.g., an enriched medical package, additional medical checkups, opportunity to be vaccinated (such as the flu) at the workplace, mental health weeks, ongoing access to a psychologist, on-site visits by a physiotherapist or nutritionist, webinars related to taking care of own health, etc.
• Support for personal development: e.g., access to webinars and workshops strengthening soft skills (such as work-life balance, working effectively from home, time management), development talks with superiors, mentoring, etc.
- Building positive relationships among employees: e.g., team-building events for the whole organization, team lunch outings, team sports events, etc.
- Activating employees in pro-social activities: e.g., supporting pro-social employee initiatives, organizing volunteer days, etc.
- Proper and supportive communication: e.g., constant availability of a supervisor, supportive feedback on work, understanding of the impact of family life or one’s own health on work results, openness to employees’ ideas and criticism, etc.

It is apparent that employees are aware of what the organization can do for increasing employee flourishing. These results also indicate that organizations are also aware and ready to take such actions. This doesn’t mean, of course, that there were no people who wrote that their organization was doing nothing in this area, or even that the atmosphere in the company was destructive to employees. However, it was a minority – about 15%. However, it is worth mentioning that in this study 28.3% of respondents answered “not at all” and “rather not” for the question “To what extent does your company care about ensuring that employees experience happiness (well-being, health) while doing their job?”. This is quite a large percentage of people participating in the study. It seems that polish organizations have a lot to improve in this area of their functioning.

## Discussion

Our study expanded the possibilities of determining flourishing employees according to the PERMA model [1]. Our research shows that the Polish version of the WPP could be useful to assess flourishing at work among Polish workers, which enables the design and implementation of further research in this group, significantly expanding the scope of existing analyses. This was achieved by examining the psychometric properties of the Polish version of the WPP. All formulated hypotheses were confirmed. The Polish version of the WPP met the reliability requirements, and the CFA indicated that the five-factor model (in contrast to the single-factor model) achieved the desired goodness-of-fit properties. The structure of the Polish version is analogous to the few other national versions. In all adaptations considering not only the translation but also the measurement of structure, relevance and reliability, a 5-dimensional structure was confirmed [18,17,19].

As expected, in addition to adequate structural validity, the Polish version of the WPP scale demonstrated convergent validity. The scale showed strong relationships with measures of positive feelings at work: PP PERMA subscales, health, overall well-being, and work satisfaction. Additionally, a negative correlation was found between the scale and negative emotions, loneliness, and perceived stress at work. In the light of the results obtained in other WPP validations, these results are analogous and confirm a certain characteristic pattern of relationships: positive relationships with other positive aspects of the employee’s functioning and negative relationships with problematic aspects.

In our research, we also used the zero-sum game as an indicator of discriminant validity which, by the way, is not a very often used procedure, but is psychometrically important [69]. The analyzes showed a statistically significant correlation with the Polish version of the WPP subscales, but they were very low (-.12 ≤ *r* ≤ -.16). Such a low level of correlation in a large sample may indicate a scale effect (a large sample gives a greater probability of significant relationships even with low indicators) rather than an actual, substantive relationship. The authors of the study accept this explanation and assume that the relationship between these variables is so low that it can be omitted. we consider the use of a measure of discriminant validity to be a strength of this study.

The Polish version of the WPP is also reliable in the context of its temporal stability. The test-retest reliability study yielded satisfactory results. The study was conducted over a two-week interval, and the findings indicated that the WPP maintained consistent scores over this period, confirming its reliability as a measurement tool.

The satisfactory psychometric properties of the Polish version of the WPP motivated us to conduct additional exploratory analyses on the relationship between employee flourishing and organizational efforts to enhance it. The moderate correlation observed between individual flourishing and organizational practices underscores the importance of employer actions in promoting employee flourish. The qualitative analysis identified several key initiatives undertaken by organizations, which align with the five causal forces shaping human experience: physical (time and form of work performance), biological (somatic health support), psychological (mental health and personal development support), and sociological (building positive relationships, pro-social activities, communication). Although most employees appreciate these efforts, a significant percentage of respondents reported insufficient organizational attention to employee flourish, indicating substantial room for improvement within Polish organizations. The quality of further research will be significantly enhanced by the ability to use the Polish version of the WPP. The questionnaire will be able to be used, for example, as an indicator of the effectiveness of organizational interventions to increase employee well-being.

The limitations of our research are closely tied to the way it is conducted. In online studies, only individuals with access to a computer can participate. Moreover, it is more challenging to control the conditions under which participants respond to questions in such situations. Another challenge associated with research conducted indirectly using online surveys is the lack of direct control of the demographic characteristics of the respondents. This may also pose some limitations to the study. However, it seems that research using the Internet, social media and other methods of remote contact with research subjects is today, and will be in the future, the main way of conducting applied research in organizations and scientific research. It seems that excessive lack of trust in respondents who could cheat about their socio-demographic characteristics does not make much sense. If someone decides to take part in the study, what motivation would he/she has to provide false data about themselves? What might be worth checking is whether completing the questionnaire electronically differs from completing it in paper-and-pencil form. However, the authors of the WPP (https://www.peggykern.org/questionnaires.html) themselves suggest the possibility of conducting research using new technologies and even indicate the method of presenting items and the scale of responses in this form of research. We followed these recommendations.

It would also be possible to improve the structure of people participating in the study so that they more literally reflect the structure of Polish society. Limitations of the study include the fact that there is a slight imbalance in gender representation in the sample (more female participants), which may be relevant and limits a little the possibility to generalize the results. Although that seems to be pretty much what happened. e.g., the proportion of women and men in Poland in 2023 was W = 51.7% and M = 48.3% (https://dbw.stat.gov.pl/baza-danych) and in this sample W = 64.5%, M = 35,2% (other, not responded = 0,3%). However, the remaining sociodemographic variables were only controlled and differ from the data from the Central Statistical Office. The analysis of the potential effect of demographic factors such as age or education is limited.

Separate concerns may arise regarding the generalizability of the findings across different work settings in Poland, given that the sample predominantly consists of corporate employees, with limited representation from smaller organizations or diverse industries. However, we would like to emphasize that similar psychometric results for the version of the WPP used in the study reported in this article were obtained in a study conducted in November–December 2024 [70]. This study focused on the flourishing of Polish teachers and specialists, including pedagogues, psychologists, and speech therapists, employed in kindergartens, primary schools, and high schools (N = 1,579). The results of this study support the five-factor model as a more suitable representation of the underlying data structure.

In subsequent research on measuring flourish at work, it would be worth taking into account the profile nature of this questionnaire. It can be assumed that in the work environment there will be larger groups of people with similar flourish results in terms of both the level of results on the scales and the configuration of these scales. Cluster analysis carried out on a large sample could allow for finding such groups. After finding such clusters, it would be possible to check how representative they are, e.g., for various organizations distinguished, for example, due to their industry affiliation.

To sum up, it should be stated that the satisfactory psychometric indicators of the WPP questionnaire and its simplicity provide many opportunities both for conducting further scientific research and for use in organizational practice.
